# *In vitro* metabolic characterization of the SARS-CoV-2 papain-like protease inhibitors GRL0617 and HY-17542

**DOI:** 10.3389/fphar.2023.1067408

**Published:** 2023-02-15

**Authors:** Hyunki Cho, Young Jun Kim, Jung-Woo Chae, Markus R. Meyer, Sang Kyum Kim, Chang Seon Ryu

**Affiliations:** ^1^ Environmental Safety Group, KIST Europe Forschungsgesellschaft mbH, Saarbrücken, Germany; ^2^ Department of Pharmacy, Saarland University, Saarbrücken, Germany; ^3^ College of Pharmacy, Chungnam National Univerisity, Daejeon, Republic of Korea; ^4^ Department of Experimental and Clinical Toxicology, Center for Molecular Signaling (PZMS), Institute of Experimental and Clinical Pharmacology and Toxicology, Saarland University, Homburg, Germany

**Keywords:** GRL0617, cytochrome P450, hepatic metabolism, LC-QTOF, human liver microsome

## Abstract

The SARS-CoV-2 pandemic requires a new therapeutic target for viral infection, and papain-like protease (Plpro) has been suggested as a druggable target. This *in-vitro* study was conducted to examine the drug metabolism of the GRL0617 and HY-17542, Plpro inhibitors. Metabolism of these inhibitors was studied to predict the pharmacokinetics in human liver microsomes. The hepatic cytochrome P450 (CYP) isoforms responsible for their metabolism were identified using recombinant enzymes. The drug–drug interaction potential mediated by cytochrome P450 inhibition was estimated. In human liver microsomes, the Plpro inhibitors had phase I and phase I + II metabolism with half-lives of 26.35 and 29.53 min, respectively. Hydroxylation (M1) and desaturation (-H2, M3) of the para-amino toluene side chain were the predominant reactions mediated with CYP3A4 and CYP3A5. CYP2D6 is responsible for the hydroxylation of the naphthalene side ring. GRL0617 inhibits major drug-metabolizing enzymes, including CYP2C9 and CYP3A4. HY-17542 is structural analog of GRL0617 and it is metabolized to GRL0617 through non-cytochrome P450 reactions in human liver microsomes without NADPH. Like GRL0617 and HY-17542 undergoes additional hepatic metabolism. The *in-vitro* hepatic metabolism of the Plpro inhibitors featured short half-lives; preclinical metabolism studies are needed to determine therapeutic doses for these inhibitors.

## 1 Introduction

**FIGURE 1:**
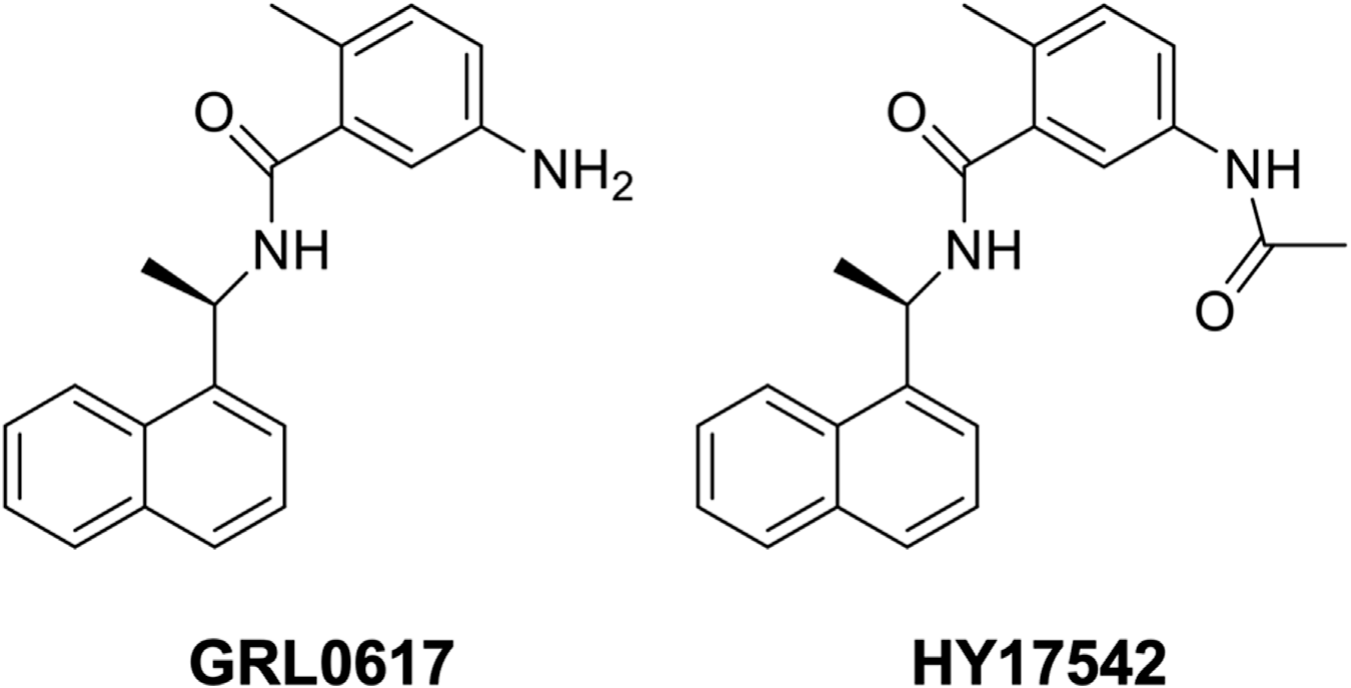
Structure of GRL0617 and HY-17542.

GRL0617 {5-amino-2-methyl-N-[(1R)-1-(1-naphthalenyl)ethyl]benzamide} was developed as a papain-like protease (Plpro) inhibitor and candidate antiviral agent (Ratia et al., 2008). HY-17542 {5-[acetylamino]-2-methyl-N-[(1R)-1-(1-naphthalenyl)ethyl]benzamide} is an acetylated form of GRL0617 (Figure 1). With the SARS-CoV-2 pandemic, GRL0617 and HY-17542 were repurposed as drug candidates for SARS-CoV-2 infection treatment (Shin et al., 2020; Fu et al., 2021), and their compatibility with SARS-CoV-2 drugs was assessed. The oral antiviral agents Paxlovid (nirmatrelvir/ritonavir) and Lagevrio (molnupiravir) were approved during the pandemic for the treatment of SARS-CoV-2 infection. Their therapeutic targets differ from those of Plpro inhibitors, and the development of drugs with new therapeutic targets is needed. Structure-based drug design has been used to improve the inhibition potency of GRL0617 and HY-17542 against SARS-CoV and SARS-CoV-2 (Ratia et al., 2008; Ghosh et al., 2009; Ghosh et al., 2010; Fu et al., 2021). However, the absorption, distribution, metabolism, excretion, and toxicology (ADMET) and pharmacokinetic proprieties of these Plpro inhibitors have not been evaluated. The aims of this study were to identify hepatic metabolites of GRL0617 and HY-17542 and evaluate their potential drug–drug interaction mediated by cytochrome P450 (CYP) inhibition. The metabolism of GRL0617 and HY-17542 was evaluated *in vitro* in human liver microsomes (HLMs) and analyzed using liquid chromatography quadrupole time-of-flight (LC-QTOF) high-resolution mass spectrometry (HRMS). In addition, CYP isoforms responsible for GRL0617 metabolism were identified using recombinant CYP enzymes. Finally, CYP inhibition assays were conducted to evaluate potential drug–drug interactions.

## 2 Materials and methods

### 2.1 Chemicals and reagents

GRL0617 (CAS no. 1093070-16-6) was obtained from Bio-Techne GmbH (Wiesbaden, Germany), and HY-17542 (1093070-14-4) was obtained from MedChemExpress LLC (Monmouth Junction, NJ, United States). Pooled HLMs (UltraPool HLM 150) were purchased from Corning (Woburn, MA, United States). Formic acid, NADPH, DMSO, and formaldehyde were purchased from Sigma Aldrich (St. Louis, MO, United States). All other reagents and chemicals were of the highest grade available commercially. Control, CYP1A2, CYP2A6, CYP2B6, CYP2C8, CYP2C9, CYP2D6, CYP2E1, CYP3A4, and CYP3A5 recombinant CYP bactosomes, carboxylesterase (CES) 1 and CES2 were purchased from Cypex (Dundee, UK). Silensome control, Silensome CYP2D6 and Silensome CYP3A4 were purchased from Biopredic international (Saint Grégoire, France).

### 2.2 *In vitro* metabolism study

#### 2.2.1 Metabolic stability assessment and metabolite identification

Microsomal incubation was conducted in triplicate using 0.1 M potassium phosphate buffer (PPB, pH 7.4) in eight-well tube strips in an 8 × 12 rack (1.2 mL; VWR, Emeryville, CA, United States). NADPH-dependent metabolism was evaluated by incubating 1 µM GRL0617 or HY-17542 with a NADPH regenerating system (1 mM NADP+, 5 mM G6P, 1 U/mL G6PDH) and 1 mg/mL pooled HLMs. The mixture was pre-incubated at 37°C with shaking at approximately 100 rpm for 5 min. UGT glucuronidation was performed with HLMs (1 mg/mL) and alamethicin (25 μg/mg microsomal protein) in a 100-mM PPB (pH 7.4) solution on ice for 15 min. After the addition of GRL0617 or HY-17542 (1 µM) and saccharic acid (1,4-lactone, 5 mM), the mixture was pre-incubated at 37°C for 5 min. CYP, UGT, and combined reactions were initiated by addition of the NADPH regenerating system, UDPGA (1 mM), and both, respectively. The reactions were quenched with 200 µL ice-cold acetonitrile containing 50 nM carbamazepine (CBZ) as an internal standard at 0, 5, 15, 30, 45, and 60 min for GRL0617, and 0, 3, 6, 10, 15, 30, and 60 min for HY-17542. The incubation mixtures were then centrifuged at 2,000 × g and 4°C for 20 min, and the supernatants were analyzed by LC-QTOF HRMS.

CYP-dependent metabolism of GRL0617 was evaluated at various concentrations (1, 3.3, 10, 25, and 50 µM) in 0.1 M PPB (pH 7.4) with 1 mg/mL HLM for 0, 5, 15, 30, 45, and 60 min incubation time. The incubation mixtures were pre-incubated at 37°C in a shaking water bath at approximately 100 rpm for 5 min. Reactions were initiated by the addition of an NADPH-regenerating system. The reaction was terminated, centrifuged then analyzed using the same method described above.

#### 2.2.2 Reaction phenotyping

Reactions were conducted in triplicate using 0.1 M PPB (pH 7.4) in eight-well tube strips in an 8 × 12 rack. The CYP isoforms responsible for metabolism were identified by incubating GRL0617 or HY-17542 (1 µM) with 50 pmol/mL of each EasyCYP which co-expressed 100 pmol/mg protein CYPs (CYP1A2, 2A6, 2B6, 2C8, 2C9, 2D6, 2E1, 3A4, 3A5, and control) together 1,000 nmol/min/mg protein cytochrome P450 reductase (CPR) in the presence of a NADPH-regenerating system at a final volume of 200 µL for 60 min. The mixture was pre-incubated at 37°C with shaking at approximately 100 rpm for 5 min. Reactions were initiated by the addition of the NADPH-regenerating system and quenched with 200 µL ice-cold acetonitrile containing 50 nM CBZ. The samples were centrifuged at 1,000 × g and 4°C for 20 min, and the supernatants were subjected to LC-QTOF HRMS. To determine the enzyme kinetic parameters, various concentrations (0, 0.22, 0.62, 1.85, 5.56, 16.67, and 50 µM) of GRL0617 were incubated in 0.1 M PPB (pH 7.4) with 50 pmol/mL of each recombinant CYP2D6, CYP3A4 or CYP3A5 enzyme. The reaction was incubated, initiated, terminated, centrifuged then analyzed using the same method described above.

#### 2.2.3 CYP inhibition assay

The drug–drug interaction potential was evaluated using a CYP cocktail assay that involved microsomal incubation and NADPH, as described elsewhere (Lee et al., 2012). Time-dependent inhibitions of CYP2C9 (tolbutamide as a selective substrate) and CYP3A4 (midazolam and testosterone as selective substrates) were evaluated using non-dilution method (Parkinson et al., 2011) with the same concentration range of GRL0617 used direct inhibition (0–50 µM) in total volume of 180 µL per tube consisting of 0.1 M PPB (pH 7.4). The first reactions were initiated by NADPH addition, and each mixture was incubated in a shaking water bath at 37°C for 30 min. The final incubation was performed for 10 min with a total volume of 200 µL by adding substrates (tolbutamide (set B) as a CYP2C9 selective substrate, midazolam (set A) and testosterone (set B) as CYP3A4 selective substrates) and NADPH. The reaction was stopped and quenched by adding 200 µL of ice-cold acetonitrile containing 50 nM CBZ as internal standard, then centrifuged at 3,000 x g for 20 min. The supernatants were injected in LC-QTOF system. IC_50_ shift values were calculated as the ratio of the IC_50_ value obtained after pre-incubation without NADPH divided by the IC_50_ value obtained after 30 min incubation with NADPH. Based on IC_50_ shifts more than 1.5-fold, the inhibitor was determined to be time-dependent inhibitor (Berry and Zhao, 2008).

#### 2.2.4 Instruments and analytical conditions

The LC-QTOF system consisted of an Exion AD LC device (AB Sciex, Framingham, MA, United States) and a TripleTOF 6,600 + system (AB Sciex) equipped with an IonDrive Turbo V source operated in positive TOF information-dependent acquisition (IDA) MS2 scan mode. The TOF scan dwell time was 50 ms, and the 20 × IDA MS2 scan time was acquired with a 30-ms dwell time. Separation was performed using a ZORBAX rapid-resolution high-definition (RRHD) Eclipse Plus C18 column (95 Å, 2.1 × 100 mm, 1.8 µm; Agilent Technologies, Santa Clara, CA, United States) coupled with an RRHD Eclipse Plus C18 guard column (2.1 mm × 5 mm, 1.8 µm; Agilent Technologies). The autosampler was operated at 4°C and the column oven was operated at 40°C, with an injection volume of 5 μL. The flow rate for mobile phases A (0.1% formic acid in water) and B (acetonitrile) was 0.3 mL/min. The initial composition of mobile phase B was 5%, which was maintained for 2 min and then increased to 20% for 2 min and 100% by 16 min, with maintenance for 2 min. The phases were then re-equilibrated at the initial condition for 2 min. The GS1, GS2, and curtain gas were set at 50, 50, 35 psi, respectively, and the ion spray voltage was 5,500 V. The collision energy for the MS2 scan was 30 eV with a 15-eV spread. CYP inhibition was assessed by detecting metabolite ions in incubated samples using the high-resolution multiple reaction monitoring (MRMhr) method with the LC-QTOF system. MRMhr transition of metabolites were described in Supplementary Table S1.

### 2.3 Statistical analysis

For all LC–MS/MS analyses, the peak areas of the parent compounds (GRL0617 and HY-17542) and metabolites were expressed as ratios to the internal standard peak areas for each test substance concentration. In the microsomal stability analysis, degradation half-life (t½) values were calculated using the following equation: t½=0.693/k where k is the first-order degradation rate constant, estimated by one-phase exponential-decay non-linear regression of the degradation time-course data using Graph Pad Prism 8.0 (Graph Pad Software Inc., San Diego, CA, United States). *In vitro* metabolic stability parameters, microsomal intrinsic clearance (CLint,mic), apparent intrinsic clearance (CL′int), and hepatic clearance (CL_h_), were calculated according to the well-stirred model approach (Pang and Rowland, 1977; Baranczewski et al., 2006). The apparent kinetic parameters of GRL0617 metabolism were determined by fitting a one-enzyme Michaelis-Menten equation. The kinetic parameters were estimated by plotting the activities over the logarithm of GRL0617 concentration with GraphPad Prism 8.0 (GraphPad Software Inc., San Diego, United States). All data are presented as mean ± SD. Data were compared between groups using the unpaired Student’s t-test. The significance level was set at *p* < 0.05, unless indicated otherwise.

## 3 Results

### 3.1 Metabolic stability and characterization of metabolic reaction of GRL0617 and HY-17542

**FIGURE 2:**
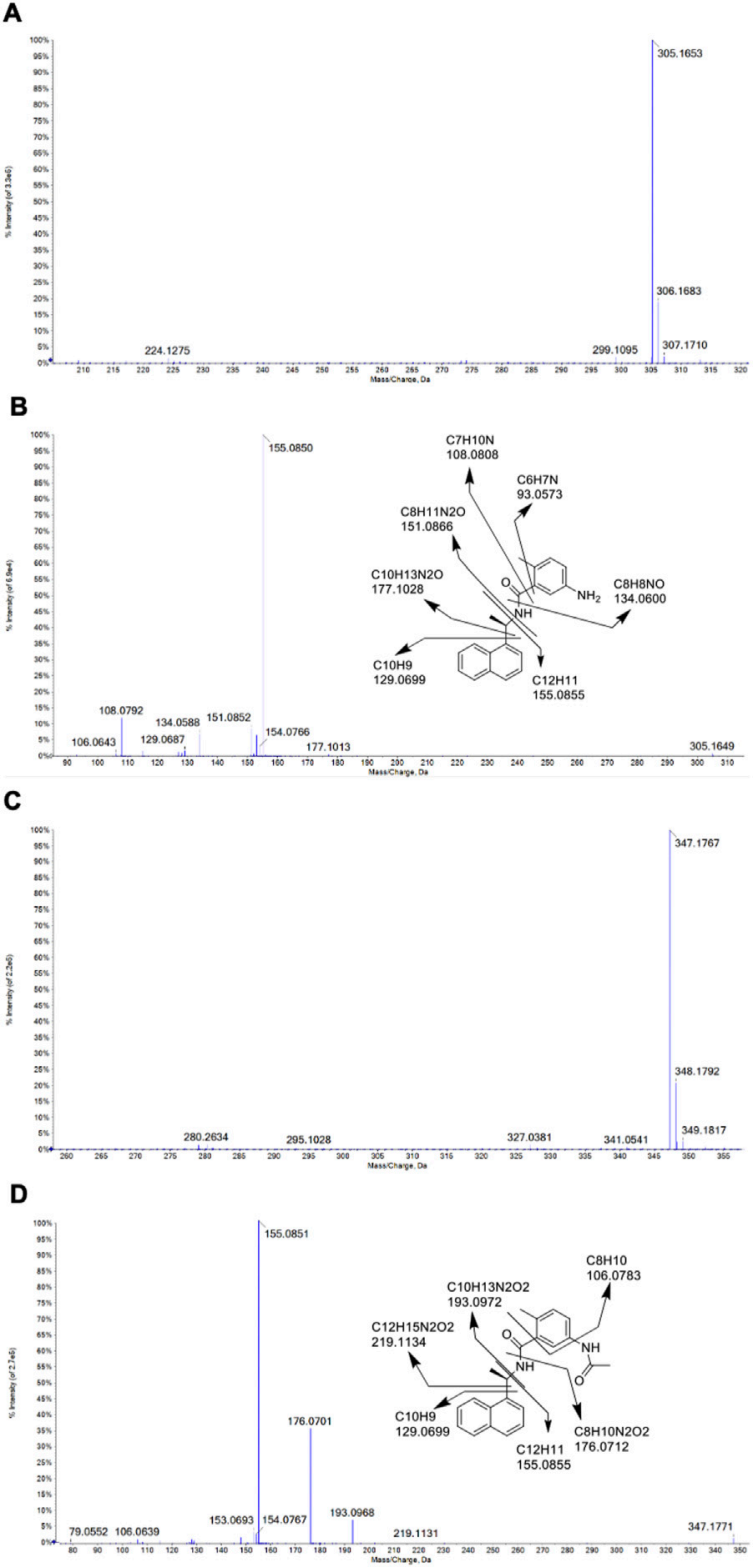
QTOF mass spectra of GRL0617 and HY-17542, obtained in the ESI + mode. **(A)** MS spectrum of GRL0617, **(B)** MS/MS spectrum and fragmentation pattern of GRL0617, **(C)** MS spectrum of HY-17542, and **(D)** MS/MS spectrum and fragmentation pattern of HY-17542.

The identities of metabolites were elucidated based on accurate mass isotope patterns in TOF MS scans and MS2 fragmentation comparisons with GRL0617 and HY-17542 standards. The chromatographic and MS fragmentation patterns of GRL0617 and HY-17542 were investigated. GRL0617 and HY-17542 eluted at 5.93 and 6.7 min, respectively, and exhibited triple protonated molecular ions at m/z 305.1563, 306.1683, and 307.1710 and 347.1767, 348.1792, and 349.1817, respectively, in positive ion mode (Figures 2A, C). The isotopic distributions of GRL0617 and HY-17542 matched well, with <5 ppm error. GRL0617 was fragmented at m/z 177.1013 (C_10_H_13_N_2_O^+^), 155.0850 (C_12_H_11_+), 151.0852 (C_8_H_11_N_2_O^+^), 134.0588 (C_8_H_8_NO^+^), 129.0687 (C_10_H_9_^+^), and 108.0792 (C_7_H_10_N^+^), and HY-17542 was fragmented at m/z 219.1131 (C_12_H_15_N_2_O_2_^+^), 193.0968 (C_10_H_13_N_2_O_2_^+^), 176.0701 (C_8_H_10_N_2_O_2_^+^), 155.0851 (C_12_H_11_^+^), 129.0699 (C_10_H_9_^+^), and 106.0639 (C_8_H_10_^+^). GRL-0617 and HY-17542 shared the C_12_H_11_ and C_10_H_9_ fragment ions containing a naphthalene ring (Figures 2B, D).

**FIGURE 3:**
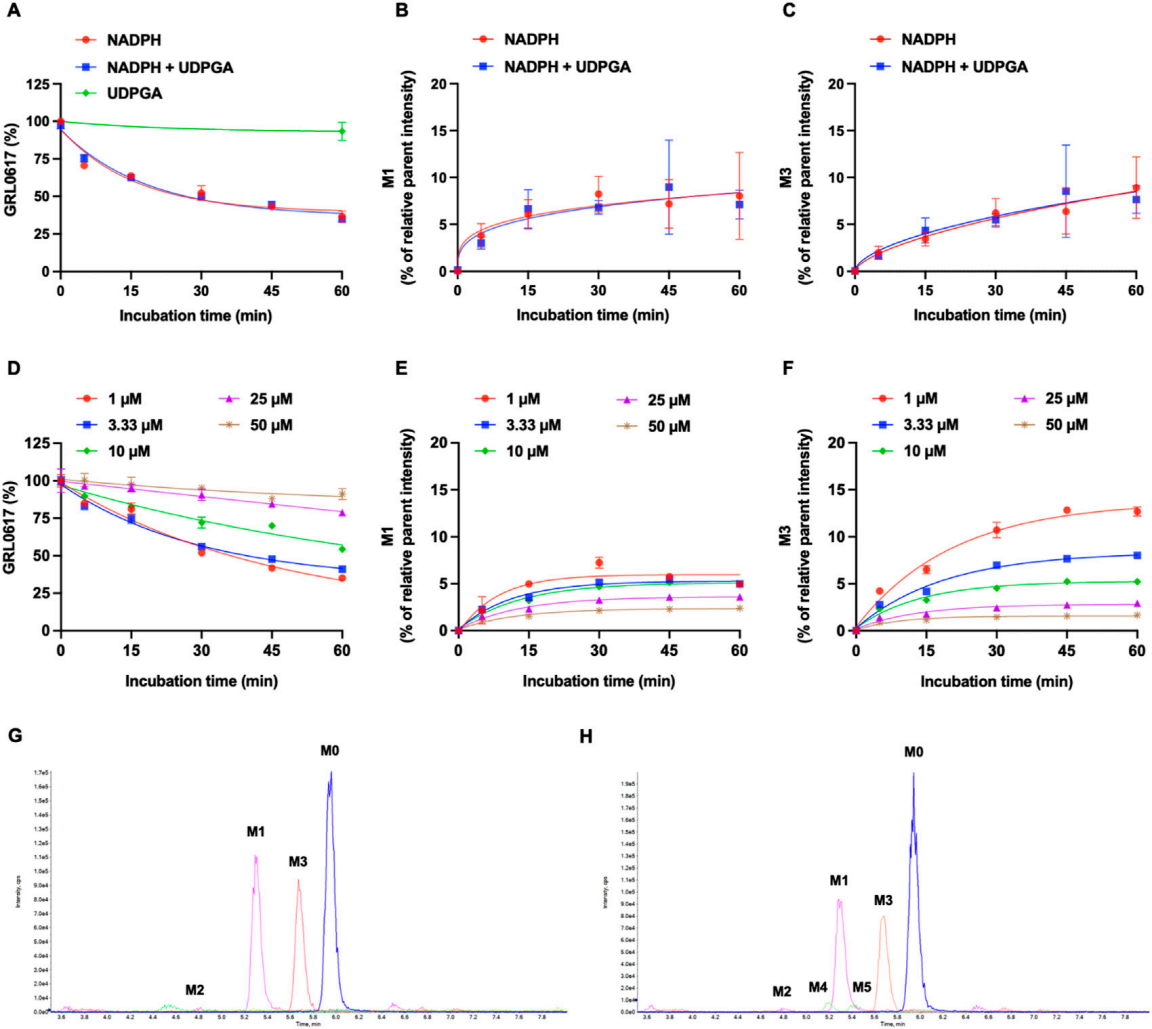
Metabolic stability of GRL0617 in HLMs. **(A)** 1 µM of GRL0617 was incubated in HLMs with NADPH, NADPH plus UDPGA, or UDPGA. **(B)** Formation of hydroxy metabolite, M1. 1 µM of GRL0617 was incubated in HLMs with NADPH, NADPH plus UDPGA. **(C)** Formation of desaturated metabolite, M3. 1 µM of GRL0617 was incubated in HLMs with NADPH, NADPH plus UDPGA. Each value represents the mean ± SD (*n* = 3). **(D)** Various concentrations (1, 3.3, 10, 25, and 50 µM) of GRL0617 were incubated in 0.1 M PPB (pH 7.4) with 1 mg/mL HLM and NADPH for 0, 5, 15, 30, 45, and 60 min. **(E)** For determination of M1 formation, various concentrations (1, 3.3, 10, 25, and 50 µM) of GRL0617 was incubated in HLMs with NADPH. **(F)** For determination of M3 formation, various concentrations (1, 3.3, 10, 25, and 50 µM) of GRL0617 were incubated in HLMs with NADPH. Data are presented as mean ± SD from three independent samples (*n* = 3). **(G)** Representative chromatogram of GRL0617 and its metabolites from QTOF MS analysis in HLMs with NADPH. **(H)** Representative chromatogram of GRL0617 and its metabolites from QTOF MS analysis in HLMs with NADPH plus UDPGA.

The NADPH- and UDPGA-dependent metabolic stability of GRL0617 (1 µM) was determined by monitoring the disappearance of the parent compound and formation of metabolites in HLMs. For reactions conducted with NADPH and NADPH + UDPGA, data were collected at six timepoints between 0 and 60 min; for those conducted with UDGPA, assessment was performed at 0 and 60 min. The GRL0617 concentration decreased to 44.5% ± 5.3%, 93.4% ± 6.1%, and 47.2% ± 7.5% of the initial GRL0617 concentration after 60 min incubation with NADPH, UDPGA, and NADPH + UDPGA, respectively (Figure 3A). No direct UGT glucuronidation was detected in HLM with UDPGA only. The calculated half-life of NADPH-dependent and NADPH + UDPGA-dependent metabolisms were 26.4 and 29.5 min, respectively.

The CLint, mic in HLM incubated with NADPH or NADPH + UDPGA was 26.3 or 23.5 μL/min/mg protein, respectively (Table 1). By *in vitro-in vivo* extrapolation of the kinetic data measured in HLM, the CL′int and CL_h_ values *in vivo* of NADPH- or NADPH + UDPGA-dependent metabolism were 27.0 and 11.7 or 24.1 and 11.1 mL/min/kg, respectively. The CL_h_ of GRL0617 was approximately half of the hepatic blood flow rate (20.7 mL/min/kg).

**TABLE 1:** Cofactor-dependent *in vitro* metabolic stability parameters of GRL0617.

Compound	Half-life	Intrinsic clearance (*in vitro*)	Intrinsic clearance (*in vivo*)	Hepatic clearance
	(min)	Cl_int_ (μL/min/mg protein)	CL_int_ (mL/min/kg)	CL_h_ (mL/min/kg)
NADPH	26.4	26.3	27.0	11.7
NADPH + UDPGA	29.5	23.5	24.1	11.1

The metabolic stability parameters were calculated according to the well-stirred model approach.

**FIGURE 4:**
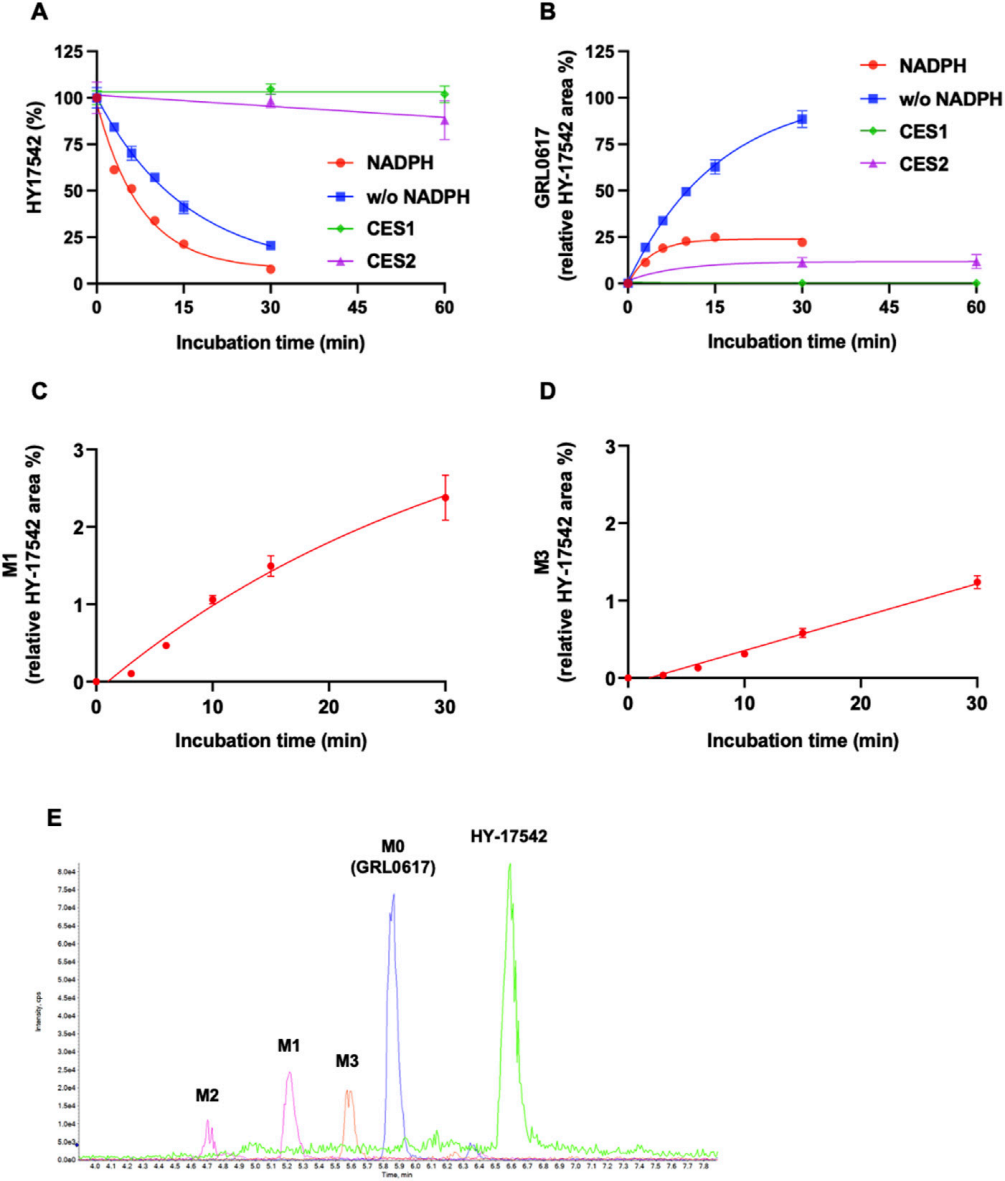
Metabolic stability of HY-17542 in HLMs. **(A)** 1 µM of HY-17542 was incubated with HLMs in the presence or absence of NADPH and incubated with recombinant CES 1 or CES2. **(B)** GRL0617 generation rates in incubations of HY-17542 with HLMs in the presence or absence of NADPH and with CES1 and CES2. **(C)** Formation of M1 in the incubation of 1 µM of HY-17542 in HLMs with NADPH. **(D)** Formation of M3 in the incubation of 1 µM of HY-17542 in HLMs with NADPH. Data are presented as mean ± SD from three independent samples (*n* = 3). **(E)** Representative chromatogram of GRL0617 and its metabolites in the presence of a NADPH-generating system from QTOF MS analysis.

Generation of M1 (hydroxylation) and M3 (desaturation, -H2), the major metabolites of GRL0617 and HY-17542, was represented in Figures 3B, C. All other generated metabolites area was less than 1% of the parent area/internal standard area. There is no detectable metabolite in HLMs incubated with UDPGA only, thus for the comparisons of the metabolic contribution of CYPs, 1–50 µM of GRL0617 was incubated in the same condition with HLM with NADPH only. The relative GRL0617 concentration and formation of M1 and M3 were presented in Figures 3D–F, respectively. M1 and M3 were the dominant metabolites of GRL0617, which is similar with the results from HLM incubated with 1 µM GRL0617. The remaining % of GRL0617 were decreased by increased GRL0617 concentration. The M1 formation in 1 µM of GRL0617 was 7.2 ± 0.6% at 30 min and decreased to 5.7 ± 0.2% at 45 min and 4.9 ± 0.2% at 60 min incubation, which was attenuated by increasing concentrations of GRL0617 (Figure 3E) M3 formation showed an increment to 45 min in all incubated concentrations (Figure 3F). The metabolic profiles of GRL0617 in HLM incubations containing NADPH and NADPH + UDPGA are shown in Figures 3G, H, respectively. HY-17542 underwent deacetylation rapidly in HLM incubated with or without NADPH (Figures 4A, B). The calculated half-lives for NADPH-dependent metabolism and NADPH-independent degradation were 5.26 and 10.83 min, respectively. We incubated HY-17542 without HLM and NADPH to 180 min in the phosphate buffer condition. HY-17542 was not significantly changed in 180 min incubation (0 min control 100 ± 1.08%, and 180 min incubation 99.89 ± 7.69, *n* = 3). CES 1 or 2 was possible enzyme responsible for the deacetylation. Recombinant CES1 did not affect HY-17542 concentration, but CES2 slightly decreased HY-17542 concentration to 88.01 ± 10.5% and produced GRL0617 from HY-17542. HY-17542 metabolites in HLM incubated with NADPH are presented in Figures 4C, D. M1 and M3 were also detected from HY-17542 in a time-dependent manner. Figure 4E shows a representative chromatogram of GRL0617 and its metabolites in HLM with NADPH from QTOF MS analysis.

M1 is hydroxylated at the para-amino toluene (also referred to as p-toluidine) side (Figure 1), with a retention time of 5.28 min. M1 displayed a hydroxylated fragment ion from the para-amino toluene side (m/z 167.0797) and a C_12_H_11_^+^ fragment ion observed at m/z 155.0837 for GRL0617 (Supplemental Figure S1A). A different fragmentation pattern was observed for M2, the other hydroxylated metabolite. The m/z 171.0768 (C_12_H_11_^+^) fragment ion corresponds to the hydroxylation of the naphthalene ring side (Supplemental Figure S1B). The M3 metabolite has a fragment ion at m/z 149.068 (C_8_H_9_N_2_O^+^), representing -H2 fragmentation of the para-amino toluene side and shared with the M1 metabolite at m/z 155.0836 (C_12_H_11_^+^), which is not changed naphthalene side ring fragment. M4 and M5 displayed the same hydroxylated fragment pattern as M1. The M1, M2, M3, M4, and M5 fragment patterns are presented in Supplemental Figure S1. Glucuronidation metabolites of the hydroxylated M4 and M5 metabolites were detected at 5.19 and 5.41 min, respectively.

### 3.2 Identification of CYPs involved in GRL0617 metabolism

**FIGURE 5:**
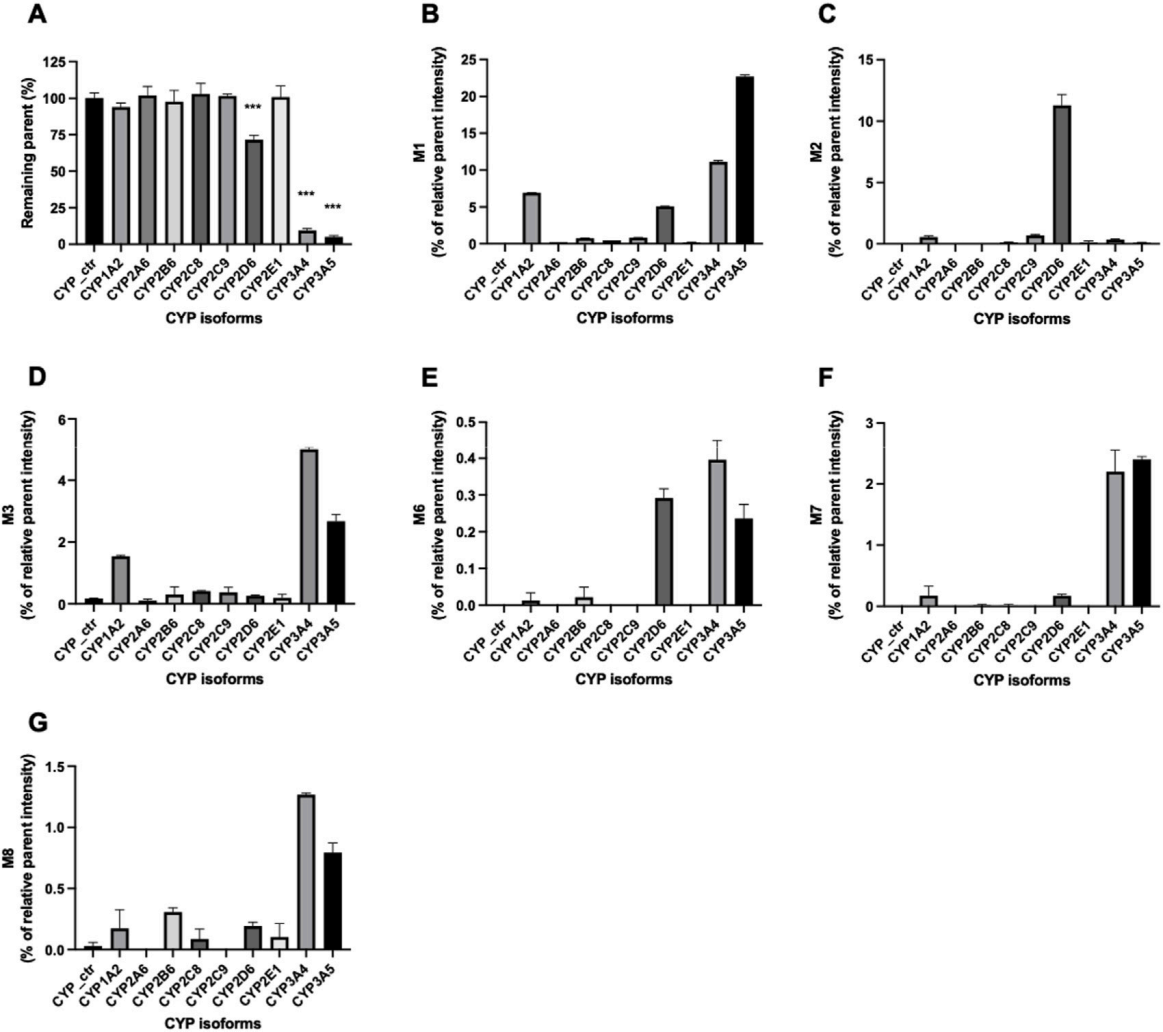
Metabolism of GRL0617 with recombinant human cytochrome P450 (CYP) enzymes. GRL0617 (1 μM) was incubated with 50 pmol/mL of each recombinant enzyme in the presence of a NADPH-generating system for 60 min. Control values were determined in a mock recombinant assay and the peak areas were compared with the control for **(A)** GRL0617 and its metabolites, including **(B)** M1, **(C)** M2, **(D)** M3, **(E)** M6, **(F)** M7, and **(G)** M8. Data are presented as mean ± SD from three independent samples (*n* = 3).

Enzymes involved in NADPH-dependent GRL0617 metabolism were determined by incubating each human recombinant CYP enzyme with GRL0617 (1 µM) for 60 min and analyzed using LC-QTOF HRMS (Figure 5). Reaction phenotyping is the identification of isoforms involved in drug metabolism and is important for the decipherment of drug biotransformation pathways and understanding of possible drug-drug interactions. Metabolites detected for GRL0617 and HY-17542 are presented in Table 2. M6, M7, and M8 were also detected in recombinant CYP incubations.

**TABLE 2:** LC-QTOF data for GRL0617, HY-17542, and their metabolites.

No.	Metabolic pathway	rt (min)	Measured [M + H]^+^	Formula	Error (ppm)
	HY-17542	6.7	347.1764	C_22_H_22_N_2_O_2_	3.7
M0	GRL0617	5.9	305.1653	C_20_H_20_N_2_O	1.5
M1	hydroxylation	5.3	321.1598	C_20_H_20_N_2_O_2_	0.3
M2	hydroxylation	4.8	321.1589	C_20_H_20_N_2_O_2_	2.6
M3	desaturation (-H_2_)	5.7	303.1487	C_20_H_18_N_2_O	1.6
M4	monooxygenation with glucuronidation	5.2	497.191	C_26_H_28_N_2_O_8_	1.7
M5	monooxygenation with glucuronidation	5.4	497.1922	C_26_H_28_N_2_O_8_	0.7
M6	two monooxygenation	4.8	337.1543	C_20_H_20_N_2_O_3_	1
M7	monooxygenation and desaturation (-H_2_)	5.3	319.1437	C_20_H_18_N_2_O_2_	1.2
M8	two monooxygenation and desaturation (-H_2_)	4.8	335.1384	C_20_H_18_N_2_O_3_	1.7

Abbreviations: rt = retention time, ppm = parts per million.

**FIGURE 6:**
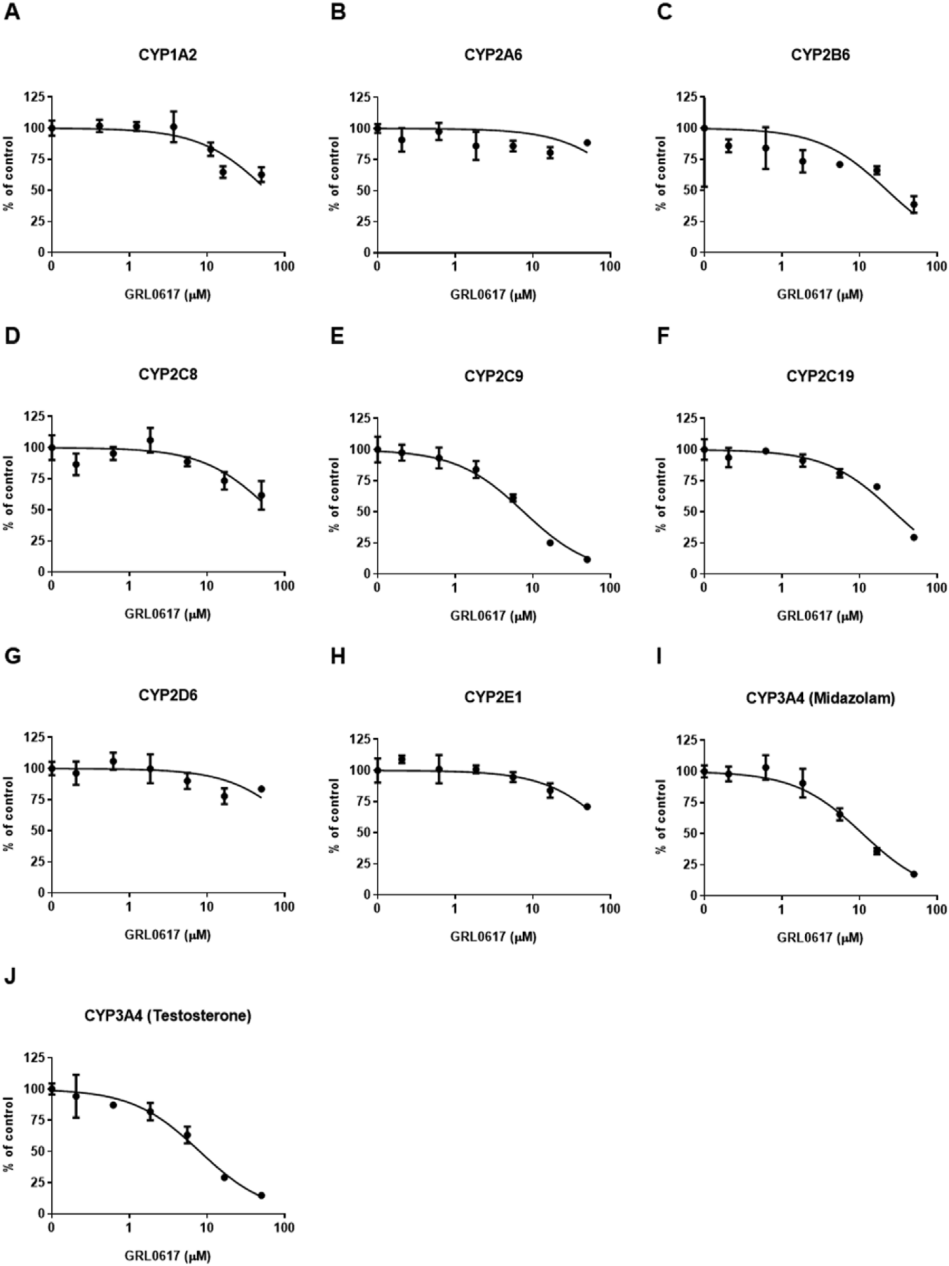
Effects of GRL0617 on the activities of **(A)** CYP1A2, **(B)** CYP2A6, **(C)** CYP2B6, **(D)** CYP2C8, **(E)** CYP2C9, **(F)** CYP219, **(G)** CYP2D6, **(H)** CYP2E1, **(I)** CYP3A4 (midazolam as a substrate), and **(J)** CYP3A4 (testosterone as a substrate) in pooled HLMs. Activity is expressed as the percentage of activity remaining as compared with a control sample containing no inhibitor (100%). Data are presented as mean ± SD from three independent samples (*n* = 3).

GRL0617 levels decreased to 71.46% ± 3.1%, 9.48% ± 1.26%, and 4.96% ± 1.08% in incubations with recombinant CYP2D6, CYP3A4, and CYP3A5 enzymes, respectively (Figure 5A). The incubation of GRL0617 with other CYP isoforms resulted in <10% reductions. Control bactosome did not affect GRL0617 remaining concentration. These results indicate that CYP2D6, CYP3A4, and CYP3A5 are involved more significantly in GRL0617 metabolism than are other isoforms. M1 formation was mediated mainly by CYP3A4 and CYP3A5, and to a lesser extent by CYP2D6 and CYP1A2 (Figure 5B). M2, the metabolite hydroxylated on the naphthalene ring side, was produced primarily with recombinant CYP2D6 enzymes. M3 is a desaturated metabolite (-2H) produced by incubation with CYP3A4 and CYP3A5, and to a lesser extent with CYP1A2 (Figure 5D). Relatively small amounts of M6, M7, and M8 metabolites were detected in incubations with CYP2D6, CYP3A4, and CYP3A5 (Figures 6E–G). Given their low abundance, the MS/MS spectra of these metabolites were not acquired.

The contribution of individual CYP isoforms to GRL0617 biotransformation in HLM was evaluated using Silensomes (Parmentier et al., 2017; Parmentier et al., 2019). Metabolic stability of GRL 0617 and formation of M1 and M3 were evaluated using Silensome control, Silensome CYP2D6 and Silensome CYP3A4 incubated with 1 or 10 µM GRL 0617 in the presence of NADPH. Other metabolites were detected less than 1% of parent. Both M1 and M3 formation was significantly reduced in Silensome CYP2D6 and CYP3A4, but CYP3A4 contribution for M1 and M3 formation was higher than CYP2D6 (Supplemental Figure S2). Enzyme kinetic analysis was performed in 50 pmol/mL recombinant CYP2D6, CYP3A4, and CYP3A5 with various concentrations of GRL0617 for 10 min (Supplemental Figure S3). The calculated parameters were presented in Table 3. CYP3A5 showed higher K_cat_/K_m_ for M1 formation than CYP3A4, but for M3 formation CYP3A4 showed higher K_cat_/K_m_ value. CYP2D6 played an exclusive role in the formation of M2, but the reactions were not saturated in the concentration range used in this study and thus, the calculated K_cat_, and K_m_ were out of the experiment condition.

**TABLE 3:** Kinetic parameters for the reactions of GRL0617 metabolism were determined using recombinants CYP2D6, CYP3A4, and CYP3A5.

	Kinetic parameters		Metabolites		
			M1	M2	M3
CYP2D6		K_m_ (µM)	85.1 (69.4–107.8)	N/A	N/A
		k_cat_ (intensity ratio/min/pmol CYP2D6)	1.4 (1.2–1.7)	N/A	N/A
		k_cat_/K_m_ (intensity ratio/min/pmol/µM)	1.7 × 10^−2^	N/A	N/A
CYP3A4		K_m_ (µM)	18.2 (14.8–22.7)	N/A	14.8 (12.3–18.0)
		k_cat_ (intensity ratio/min/pmol CYP3A4)	1.7 (1.6–1.9)	N/A	0.36 (0.34–0.39)
		k_cat_/K_m_ (intensity ratio/min/pmol CYP3A4/µM)	9.6 × 10^−2^	N/A	2.4 × 10^−2^
CYP3A5		K_m_ (µM)	8.5 (7.6–9.5)	N/A	8.5 (11.6–16.7)
		k_cat_ (intensity ratio/min/pmol CYP3A5)	1.4 (12.43–16.7)	N/A	0.22 (0.20–0.23)
		k_cat_/K_m_ (intensity ratio/min/pmol CYP3A5/µM)	1.7 × 10^−1^	N/A	1.6 × 10^−2^

GRL0617 (0, 0.22, 0.62, 1.85, 5.56, 16.67, and 50 µM) was incubated with each recombinant CYP, enzyme in the presence of an NADPH, generating system for 10 min. The apparent Km and Kcat values were calculated by non-linear regression using the Michaelis-Menten equation. Data are expressed as means ± SD, for three independent samples. Values in parentheses represent 95% confidential intervals. N/A, not applicable.

In addition, the contribution of specific isoform using Silensomes were evaluated, and calculated parameters were shown in Supplemental Figure S4 and Table 4. Half-life of GRL0617 in the Silensome control, Silensome CYP2D6 or Silensome CYP3A4 was calculated as 17.4, 22.3, and 85.1 min, respectively. The contribution of specific CYP 3A4 or CYP2D6 was calculated to be 79.6% or 21.8%, respectively, using equation as equation below. Calculated % CYP isoform contribution = CLint_control_−Clint_INH_ ⁄ CLint_control_ (Bohnert et al., 2016).

**TABLE 4:** Contribution of CYP2D6 and CYP3A4 to GRL0617 *in vitro* metabolism in Silensomes.

Factor	Half-life	Intrinsic clearance (*in vitro*)	Contribution
	(min)	Cl_int_ (μL/min/mg protein)	(%)
Silensome control	17.4	39.8	100
Silensome CYP2D6	22.3	31.2	21.8
Silensome CYP3A4/3A5	85.1	8.14	79.6

### 3.3 Effect of GRL0617 on CYP isoform activity in HLMs

An LC-MS/MS–based CYP inhibition assay was performed using CYP substrates (Lee and Kim, 2013). The ability of GRL0617 to inhibit the formation of metabolites by nine CYP isoforms (CYP1A2, 2A6, 2B6, 2C8, 2C9, 2C19, 2D6, 2E1, and 3A4) was evaluated using pooled HLMs and substrate cocktails (Figure 6). Experimental GRL0617 IC_50_ values were determined from seven-point concentration-response curves. Ketoconazole was used as a positive control with strong CYP3A4 inhibition ability and an IC_50_ value within the reference range (data not shown).

Tolbutamide 4-hydroxylation formation was used to determine CYP2C9 activity and midazolam 1-hydroxylation and testosterone 6β-hydroxylation formation were taken to indicate CYP3A4 activity; both activities were markedly inhibited (Figures 6E, I, J). GRL0617 IC_50_ values with 95% confidence intervals (CIs) were 7.6 μM (6.3–9.2 μM) for CYP2C9, 10.9 μM (8.7–13.6 μM) for CYP3A4 (midazolam), and 8.0 μM (6.4–10.1 μM) for CYP3A4 (testosterone). GRL0617 weakly inhibited CYP1A2 [IC_50_ = 59.6 μM (42.9–82.7 μM)], CYP2B6 [IC_50_ = 22.6 μM (11.0–46.2 μM)], and CYP2C19 [IC_50_ = 27.5 μM (22.4–33.8 μM)] relative to CYP2C9 and CYP3A4. GRL0617 inhibited 2A6, 2D6, and CYP2E1 at concentrations ≥ 50 μM (Figures 6B, G, H).

The time-dependent inhibition of CYP2C9 and CYP3A4 by GRL0617 showing relatively strong inhibitory effect than other isoforms were evaluated using IC_50_ shifts between microsomes incubated with and without NADPH for 30 min pre-incubation. Shifts of GRL0617 IC_50_ values against CYP2C9 using 100 μM tolbutamide as a substrate and CYP3A4 using 5 μM midazolam and 50 μM testosterone were determined from seven-points concentration-response curves. The IC_50_ shifts of GRL0617 on CYP2C9, CYP3A4 (midazolam), and CYP3A4 (testosterone) were 3.38, 10.8, and 1.54, respectively. The results suggest that the GRL0617-induced CYP inhibition may be time-dependent.

## 4 Discussion

In January 2022, more than 660 million people had confirmed COVID-19; more than 6.7 million COVID-19–related deaths and 13 million vaccinated individuals were reported (WHO, 2023). Paxlovid (nirmatrelvir/ritonavir) and Lagevrio (molnupiravir) are approved for the treatment of COVID-19. Nirmatrelvir is a SARS-CoV-2 main protease (also known as 3CL protease) inhibitor, and molnupiravir promotes widespread mutation during the replication of viral RNA by RNA-directed RNA polymerase (Kabinger et al., 2021). Molnupiravir has lower efficacy to treat COVID-19 than Paxlovid (Saravolatz et al., 2022). With the emergence of numerous SARS-CoV-2 variants, concern about acquired viral resistance to nirmatrelvir has arisen (Iketani et al., 2022; Yang et al., 2022). Thus, additional targeted inhibitors are required for SARS-CoV-2 infection treatment. In 2008, GRL0617 was found to be a Plpro inhibitor (Ratia et al., 2008); subsequent structural studies have led to the identification of new Plpro inhibitors with improved anti–SARS-CoV-2 potency (Elseginy and Anwar, 2021; Rao et al., 2022; Shen et al., 2022). The metabolic stability of these analog compounds has been reported only in mouse microsomes (Báez-Santos et al., 2014), and as *in silico* predictions (Parmar et al., 2021; Rao et al., 2022). *In vitro* experimental investigations of the metabolites, reaction phenotyping, and CYP inhibition have not been reported for GRL0617.

Hepatic drug metabolism can produce metabolites whose pharmacological activities differ from those of the parent drugs. *In vitro* assays have shown that GRL0617 acts more efficiently against SARS-CoV and SARS-Cov-2, with an IC_50_ value approximately five-fold lower than that of HY-17542 (Ratia et al., 2008; Fu et al., 2021). HY-17542 undergoes rapid deacetylation to GRL0617, and thus can be used as a GRL0617 prodrug as long as it has more advantageous physicochemical properties than does GRL0617. The halflife of GRL0617 during phase I metabolism was <30 min, indicating very high clearance of GRL0167 and HY-17542. To maintain sufficient plasma concentrations, additional pharmacokinetic studies and the development of new GRL0617 analogs should be undertaken.

The *in silico* prediction tool pkCSM indicated that GRL0617 is a CYP3A4 substrate, but not a CYP2D6 substrate (Rao et al., 2022). GRL0617 was also predicted to inhibit CYP1A2, CYP2C9, CYP2C19, and CYP3A4, but not CYP2D6 (Rao et al., 2022). In the present study, CYP3A4, CYP3A5, and CYP2D6 contributed to the elimination of GRL0617 in HLM incubations. We determined contribution of CYP3A4 and CYP2D6 in using Silensome and CYP3A4 has bigger contribution than CYP2D6 for M1 and M3 metabolism. As GRL0617 showed the time-dependent manner of CYP3A4 inhibition, which is also involved in its metabolism, long-term treatment may have the effect of further increasing GRL0617 concentrations in plasma through CYP3A4 inhibition. Further precise kinetic experiments with specific reaction with quantification is required for pharmacokinetic prediction and the estimation of drug–drug interactions.

CYP3A4/5 metabolizes various chemicals composed of large, lipophilic molecules with diverse structures, and >50% of all prescribed drugs undergo CYP3A4 metabolism (Zanger and Schwab, 2013). CYP3A4-related drug interaction studies are critical for the prediction of drug–drug interactions in the clinic (Prueksaritanont et al., 2013). The current study showed that GRL0617 concentrations were lower in CYP3A5 incubations than in other CYP isoforms, and M1 formation was higher CYP3A5 than CYP3A4. In addition, Silensome CYP3A4 was prepared using pre-treatment with azamulin that also inhibits CYP3A5 (Stresser et al., 2004; Parmentier et al., 2017). Thus, Silensome 3A4 represented contribution of both CYP3A4 and CYP3A5, compared with control Silensome. CYP3A5 expression in the liver differs between Africans/African Americans and Caucasians. Like the immune suppressor tacrolimus, CYP3A5 expressor genotypes (CYP3A5*1/* and *1/*3) may contribute significantly to GRL0617 metabolism in individuals with low CYP3A4 expression (Lamba et al., 2012). CYP2D6 is a highly polymorphic gene involved in drug metabolism (Ingelman-Sundberg et al., 2007). Its ultrarapid metabolizer phenotypes, such as those possessing copy number variants CYP2D6*1 and CYP2D6*2, may also contribute significantly to GRL0617 metabolism. Indeed, CYP2D6 metabolized the naphthalene side ring through hydroxylation, which is not linked to M1–M3 dehydration.

GRL0617 hydroxylation was observed on the para-amino toluene and ethyl-naphthalene side chains. Desaturation (-H2) of the para-amino toluene side chain might produce an imine methide, a reactive and toxic metabolite. The aromatic amine moiety on GRL0617 raises concerns about toxicity, including carcinogenesis and erythrocyte lysis (Limban et al., 2018). Piperidine-4-carboxamide scaffold-substituted compounds based on GRL0617 were developed to improve efficacy against SARS-CoV; however, the compounds were metabolically unstable in mouse liver microsomes (Báez-Santos et al., 2014). These metabolic features should be considered with the development of GRL0617 derivative compounds.

**FIGURE 7:**
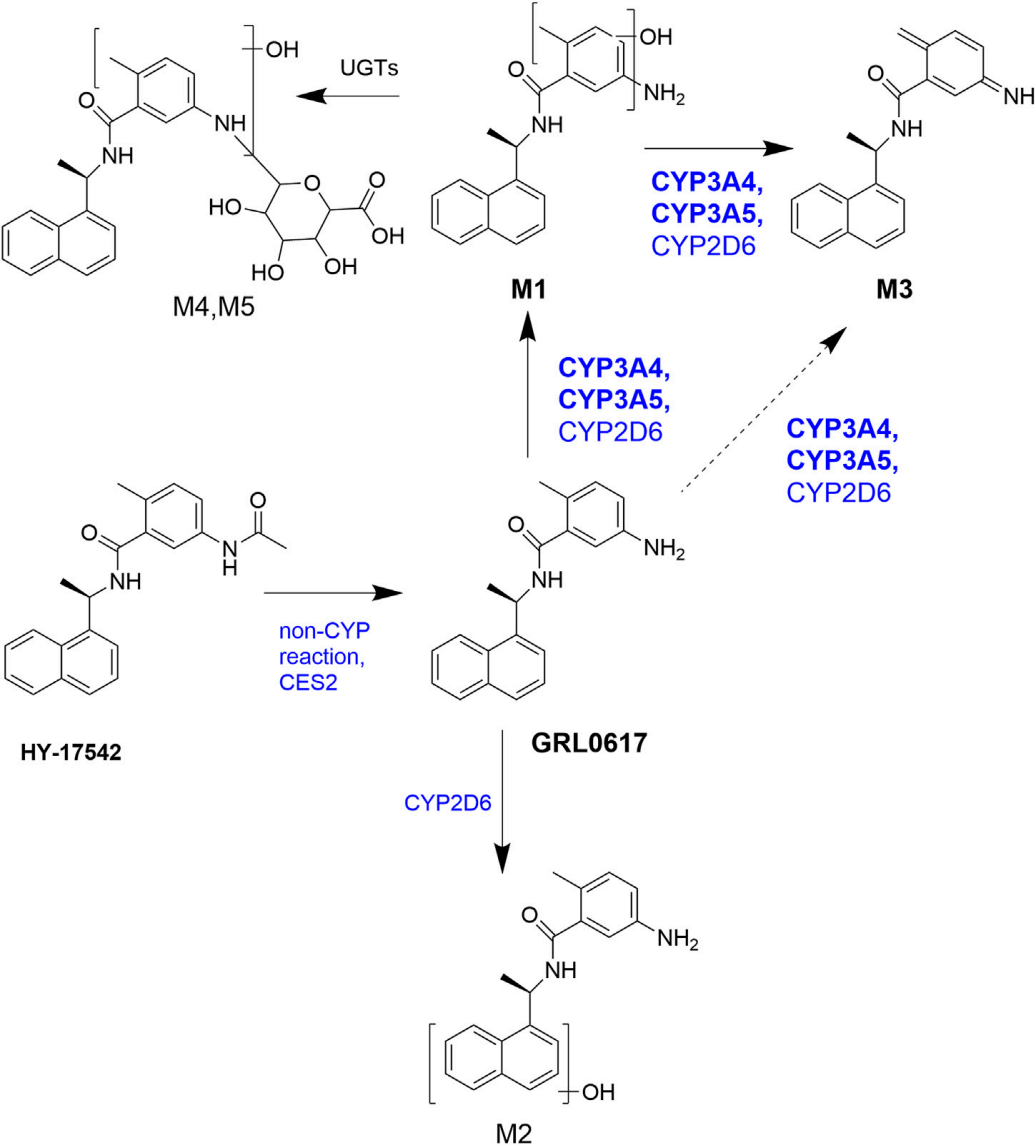
Proposed metabolic pathways of GRL0617 and HY-17542 in the liver.

The proposed metabolism of GRL0617 and HY-17542 is presented in Figure 7. In summary, we suggest that CYP enzymes play major roles in hepatic GRL0617 and HY-17542 metabolism. HY-17542, an acetylated analog of GRL0617, is metabolized rapidly to GRL0617. Our experiments with recombinant enzymes showed that CYP3A4, CYP3A5, and CYP2D6 are involved in GRL0617 elimination and the formation of hydroxylated metabolites M1, M2, and M3. CYP3A4/5 played a dominant role in the hydroxylation and -H2 oxidation of para-amino toluene groups. GRL0617 also inhibits CYP3A4 and CYP2C9. Further research is required to assess GRL0617s toxicity, mechanism of CYP inhibition and clearance in hepatocytes.

## Data Availability

The raw data supporting the conclusion of this article will be made available by the authors, without undue reservation.
