# Supplementary material for: In vitro metabolic characterization of the SARS-CoV-2 papain-like protease inhibitors GRL0617 and HY-17542
Source: Front Pharmacol. 2023 Feb 15;14:1067408. doi: 10.3389/fphar.2023.1067408 (PMC9975351; doi:10.3389/fphar.2023.1067408)
Supplement: Supplementary file 1 [file DataSheet1.pdf]

Supplementary Material

In vitro metabolic characterization of the SARS-CoV-2 papain-like protease inhibitors GRL0617 and HY-17542

Hyunki Cho[1,2], Young Jun Kim[1], Jung-woo Chae[3], Markus R. Meyer[4], Sang Kyum Kim[3*], Chang Seon Ryu[1*]

[1]Environmental Safety Group, KIST Europe Forschungsgesellschaft mbH, 66123 Saarbrücken, Germany

2Department of Pharmacy, Saarland University, Saarbrücken, Germany

[3]College of Pharmacy, Chungnam National Univerisity, 34134 Daejeon, Republic of Korea

[4]Department of Experimental and Clinical Toxicology, Institute of Experimental and Clinical Pharmacology and Toxicology, Center for Molecular Signaling (PZMS), Saarland University, Homburg, Germany

*** Correspondence:**
Sang Kyum Kim
sangkim@cnu.ac.krs

Chang Seon Ryu
changryu@kist-europe.de

Supplementary Material

Supplement Table 1. MRMhr transition of specific CYP substrates.

CYP isoforms	Name	Chemical Formula	Adduct/ Charge	Precursor (Q1) Mass (Da)	Fragment (Q3) Mass (Da)
CYP1A2	Acetaminophen	C8H9NO2	[M+H]+	152.0706	110.0588
CYP2A6	7-Hydroxy coumarin	C9H6O3	[M+H]+	163.039	107.0481
CYP2B6	Hydroxybupropion	C13H18ClNO2	[M+H]+	256.1099	238.0995
CYP2C8	Desethylamodiaquine	C18H18ClN3O	[M+H]+	328.1211	283.0649
CYP2C9	4-Hydroxy tolbutamide	C12H18N2O4S	[M+H]+	287.106	269.1897
CYP2C19	4-Hydroxy mephenytoin	C12H14N2O3	[M+H]+	235.1077	150.091
CYP2D6	Dextrorphan	C17H23NO	[M+H]+	258.1852	157.0642
CYP3A4	6β-hydroxy testosterone	C19H28O3	[M+H]+	305.2111	269.171
CYP3A4	1-Hydroxy midazolam	C18H13ClFN3O	[M+H]+	342.0804	324.071
Internal standard	Carbamazepine	C15H12N2O	[M+H]+	237.1022	194.0963

A

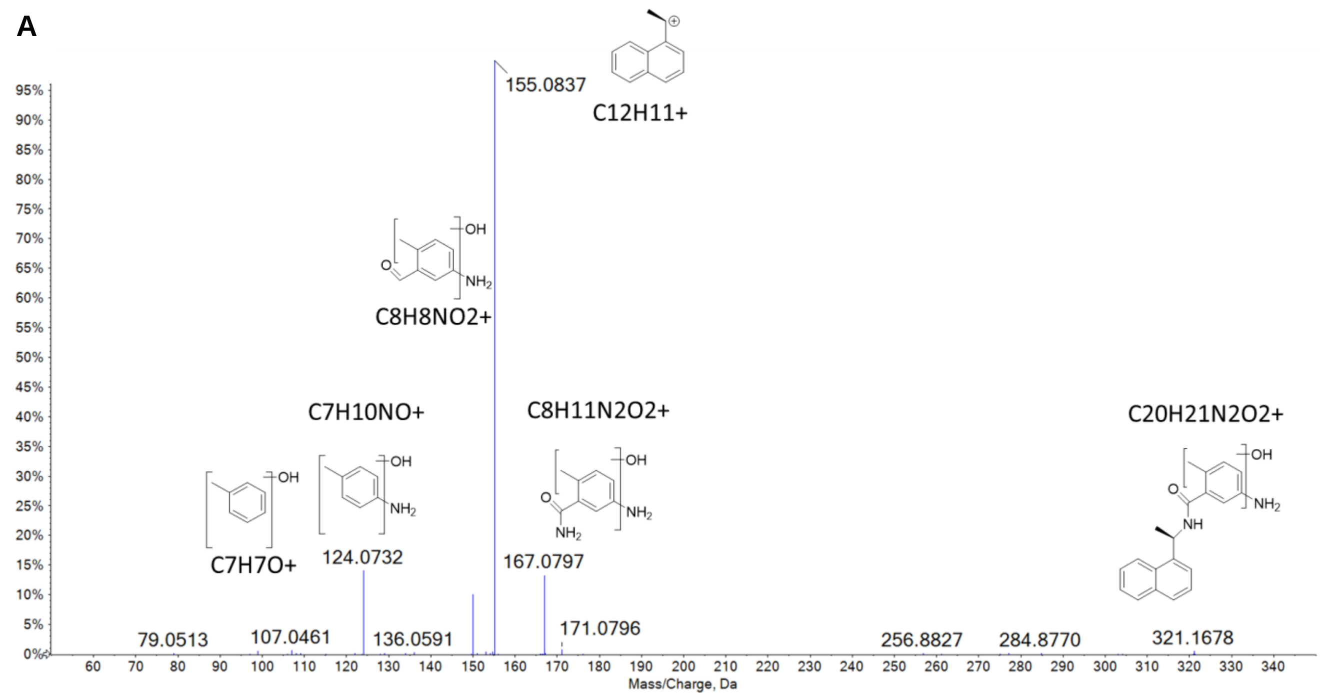
155.0837
C12H11+
C8H8NO2+
C7H10NO+
C8H11N2O2+
C20H21N2O2+
C7H7O+
124.0732
167.0797
79.0513
107.0461
136.0591
171.0796
256.8827
284.8770
321.1678
Mass/Charge, Da

B

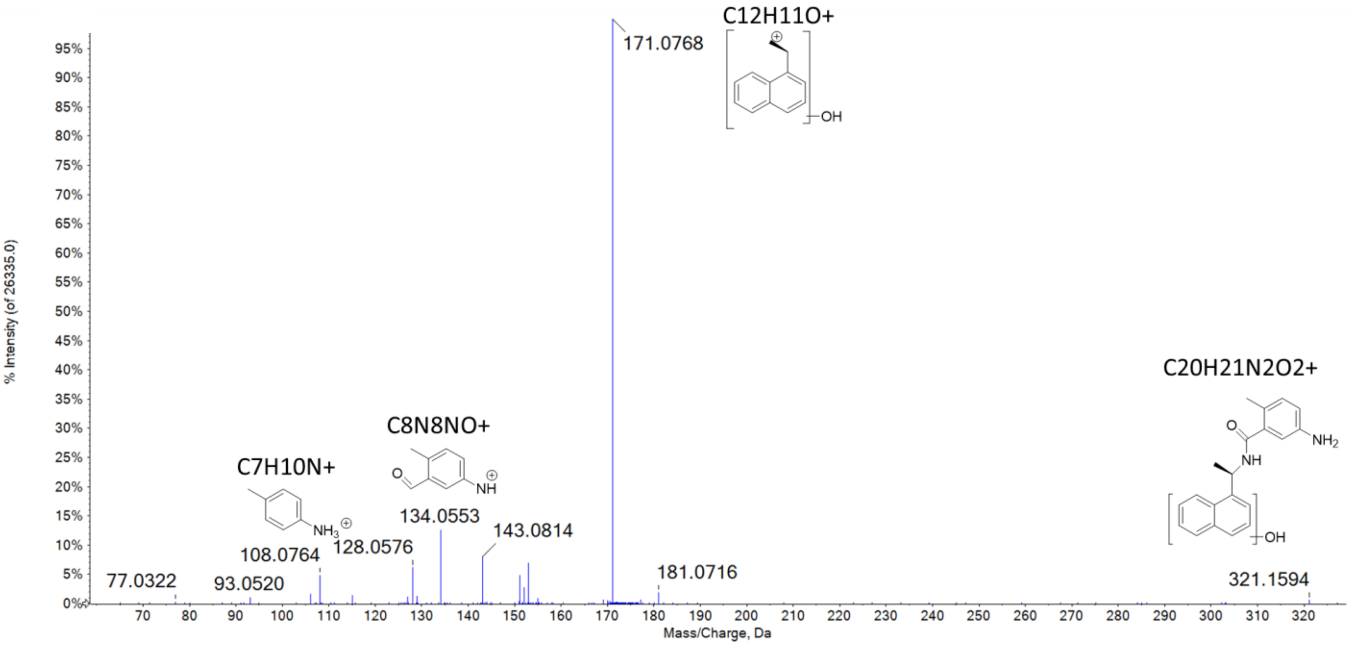
171.0768
C12H11O+
% Intensity (of 26335.0)
C20H21N2O2+
C8N8NO+
C7H10N+
134.0553
143.0814
108.0764
128.0576
77.0322
93.0520
181.0716
321.1594
Mass/Charge, Da

C

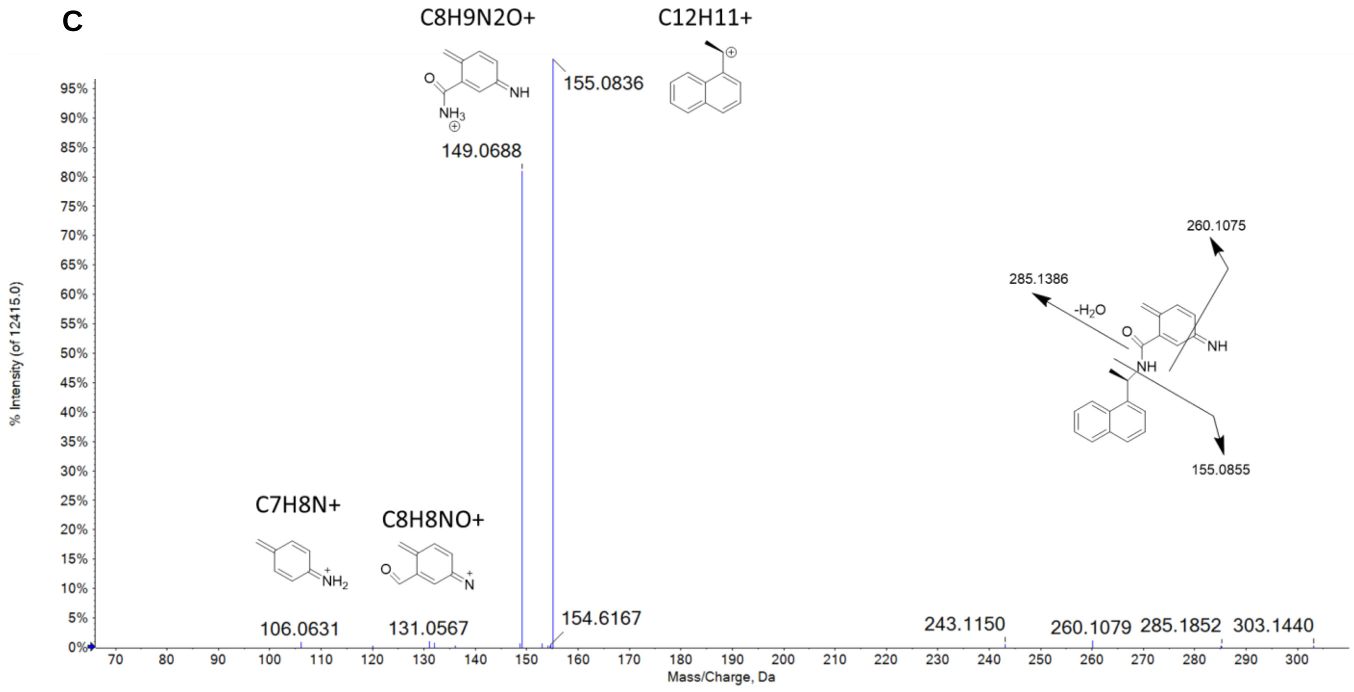
C8H9N2O+
C12H11+
155.0836
149.0688
260.1075
285.1386
-H2O
155.0855
C7H8N+
C8H8NO+
106.0631
131.0567
154.6167
243.1150
260.1079
285.1852
303.1440
% Intensity (of 12415.0)
Mass/Charge, Da

D

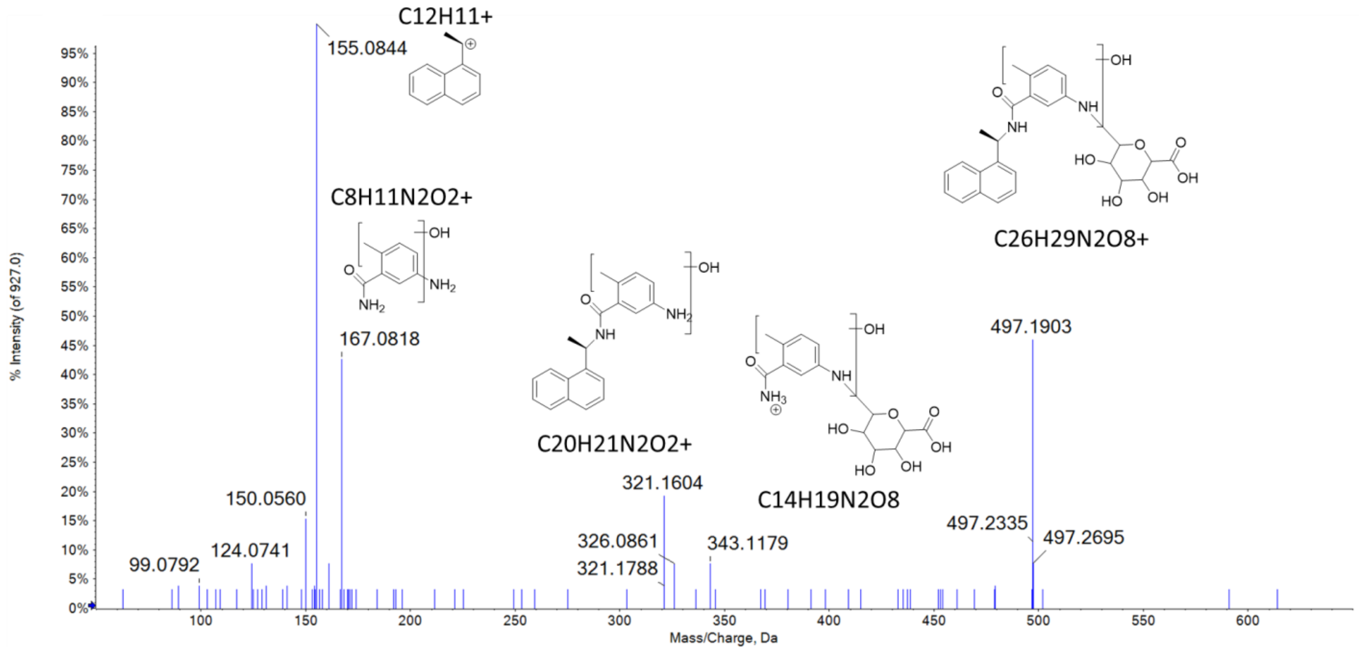
C12H11+
155.0844
C8H11N2O2+
167.0818
C26H29N2O8+
C20H21N2O2+
C14H19N2O8
497.1903
321.1604
150.0560
326.0861
343.1179
321.1788
497.2335
497.2695
124.0741
99.0792
% Intensity (of 927.0)
Mass/Charge, Da

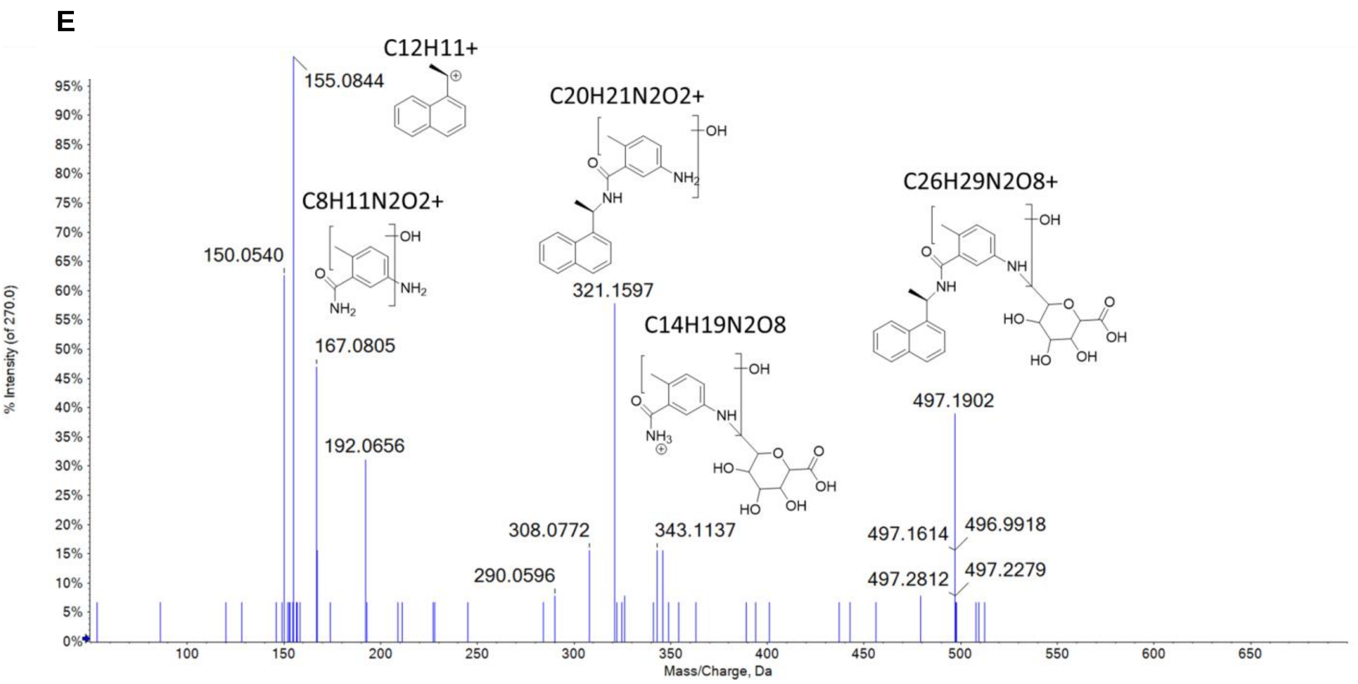

Supplementary Figure 1. MS/MS spectra and fragmentation patterns of GRL0617 metabolites. (A) M1, (B) M2, (C) M3, (D) M4, and (E) M5.

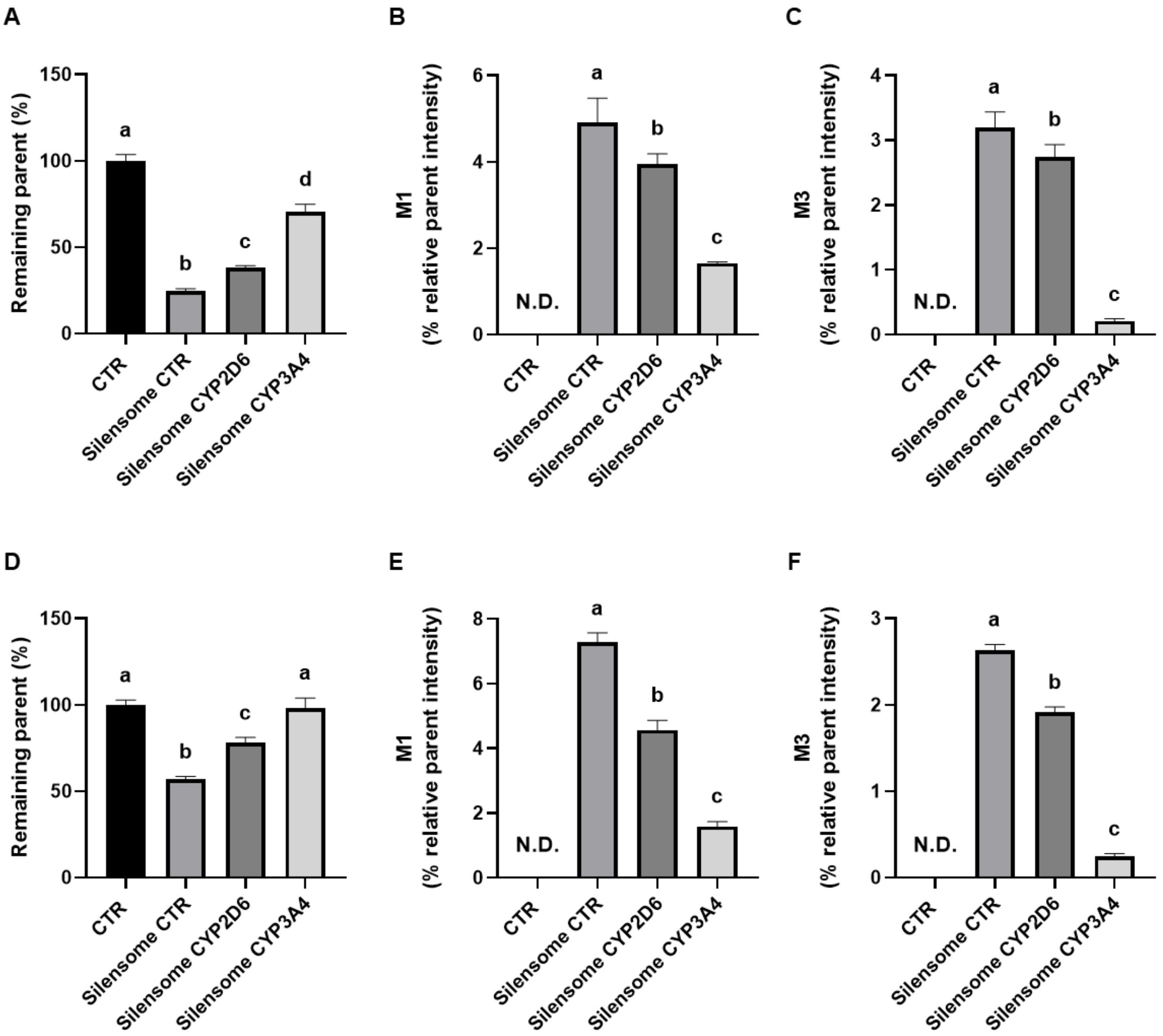

Supplementary Figure 2. Effect of specific CYP isoform inhibitors on the metabolism of GRL0617 using Silensome. (A-C) 1 µM of GRL0617 was incubated in Silensome control without NADPH (CTR) or Silensome control, Silensome CYP2D6, and Silensome CYP3A4 with NADPH for 60 min. (A) Remaining GRL0617, (B) formation of M1, and (C) formation of M3. (D-F) 10 µM of GRL0617 was incubated in Silensome control without NADPH (CTR) or Silensome control, Silensome CYP2D6 and Silensome CYP3A4 with NADPH. (D) Remaining GRL0617, (E) formation of M1, and (F) formation of M3. Each value represents the mean ± SD (n = 3). Statistical differences between control and exposed groups were analyzed by one-way analysis of variance ANOVA followed by Tukey's multiple comparison tests at $P < 0.05$.

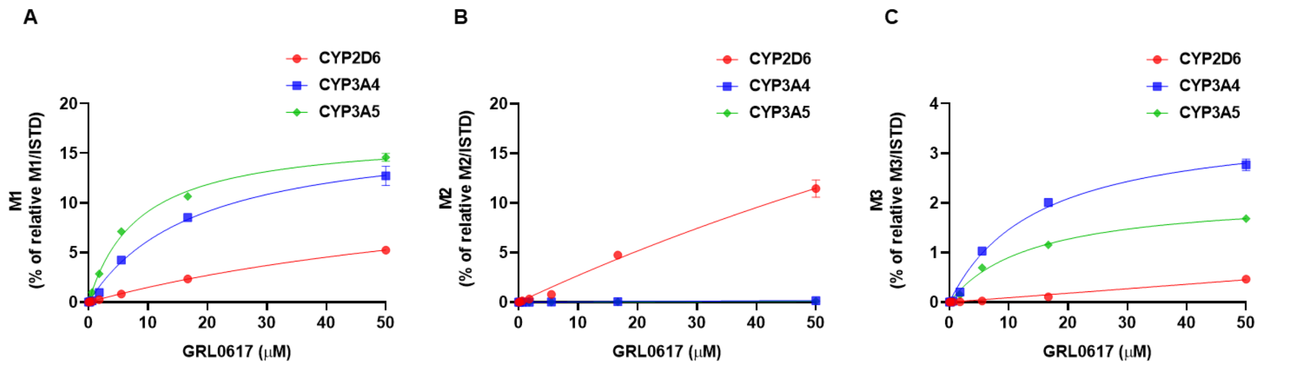

Supplementary Figure 3. Enzyme kinetic analysis was performed in 50 pmol/mL recombinant CYP2D6, CYP3A4, and CYP3A5 with various concentrations (0, 0.22, 0.62, 1.85, 5.56, 16.67, and 50 μM) of GRL0617 for 10 min. (A) Formation of M1, (B) formation of M2, and (C) formation of M3. The relative peak area ratios of metabolites divided by the internal standard are expressed as mean ± SD (*n*=3).

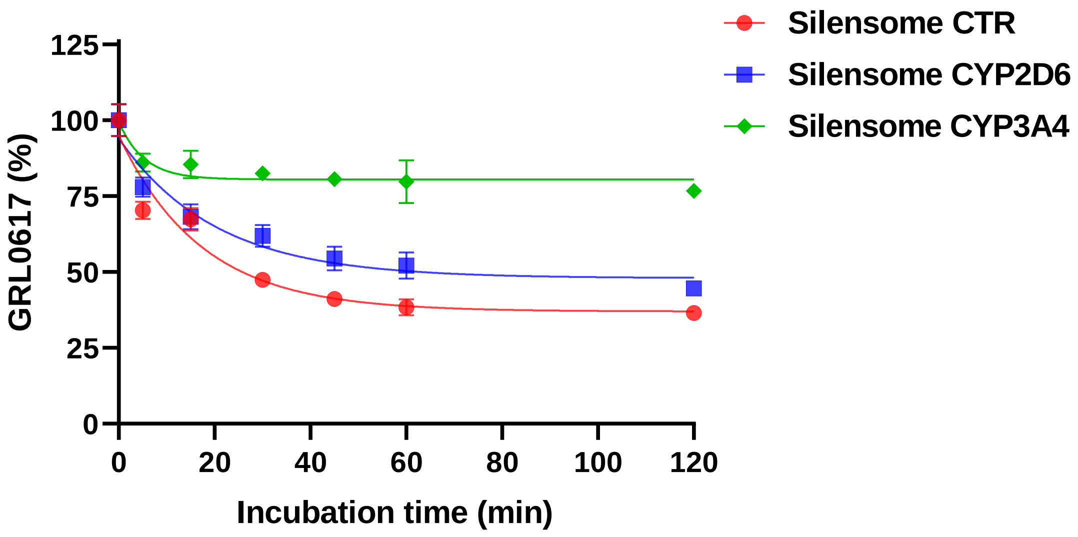

Supplementary Figure 4. Metabolic stability of GRL0617 in Silensome. (A) 1 µM of GRL0617 was incubated in Silensome control, Silensome CYP2D6, and Silensome CYP3A4 with NADPH for 0, 10, 15, 30, 45, 60, or 120 min. The remaining percentages of GRL 0617 are expressed as mean ± SD (*n*=3).
